# Identification and Validation of Stable Quantitative Trait Loci for SDS-Sedimentation Volume in Common Wheat (*Triticum aestivum* L.)

**DOI:** 10.3389/fpls.2021.747775

**Published:** 2021-12-07

**Authors:** Shuai Tian, Minghu Zhang, Jinghui Li, Shaozhe Wen, Chan Bi, Huanhuan Zhao, Chaoxiong Wei, Zelin Chen, Jiazheng Yu, Xintian Shi, Rongqi Liang, Chaojie Xie, Baoyun Li, Qixin Sun, Yufeng Zhang, Mingshan You

**Affiliations:** ^1^State Key Laboratory for Agrobiotechnology, Key Laboratory of Crop Heterosis and Utilization, The Ministry of Education, Key Laboratory of Crop Genetic Improvement, China Agricultural University, Beijing, China; ^2^Wheat Center, Henan Institute of Science and Technology, Henan Provincial Key Laboratory of Hybrid Wheat, Xinxiang, China; ^3^National Plant Gene Research Centre, Beijing, China

**Keywords:** SDS-sedimentation volume, major QTL, wheat, quality, mapping

## Abstract

Sodium dodecyl sulfate-sedimentation volume is an important index to evaluate the gluten strength of common wheat and is closely related to baking quality. In this study, a total of 15 quantitative trait locus (QTL) for sodium dodecyl sulfate (SDS)-sedimentation volume (SSV) were identified by using a high-density genetic map including 2,474 single-nucleotide polymorphism (SNP) markers, which was constructed with a doubled haploid (DH) population derived from the cross between Non-gda3753 (ND3753) and Liangxing99 (LX99). Importantly, four environmentally stable QTLs were detected on chromosomes 1A, 2D, and 5D, respectively. Among them, the one with the largest effect was identified on chromosome 1A (designated as *QSsv.cau-1A.1*) explaining up to 39.67% of the phenotypic variance. Subsequently, *QSsv.cau-1A.1* was dissected into two QTLs named as *QSsv.cau-1A.1.1* and *QSsv.cau-1A.1.2* by saturating the genetic linkage map of the chromosome 1A. Interestedly, favorable alleles of these two loci were from different parents. Due to the favorable allele of *QSsv.cau-1A.1.1* was from the high-value parents ND3753 and revealed higher genetic effect, which explained 25.07% of the phenotypic variation, mapping of this locus was conducted by using BC_3_F_1_ and BC_3_F_2_ populations. By comparing the CS reference sequence, the physical interval of *QSsv.cau-1A.1.1* was delimited into 14.9 Mb, with 89 putative high-confidence annotated genes. SSVs of different recombinants between *QSsv.cau-1A.1.1* and *QSsv.cau-1A.1* detected from DH and BC_3_F_2_ populations showed that these two loci had an obvious additive effect, of which the combination of two favorable loci had the high SSV, whereas recombinants with unfavorable loci had the lowest. These results provide further insight into the genetic basis of SSV and *QSsv.cau-1A.1.1* will be an ideal target for positional cloning and wheat breeding programs.

## Introduction

Common wheat is one of the most widely cultivated food crops in the world and one of the important sources of carbohydrates and proteins for human beings (Osinowo, 2011). For a long time, breeders of common wheat (*Triticum aestivum* L.) have mainly committed to improving wheat yield and disease resistance (Curtis and Halford, 2014). However, high-quality wheat has been demanded by consumers and industries, and wheat quality improvement has attracted increasing attention among breeders (Guzman et al., 2016). Gluten strength has a considerable influence on the end-use quality of wheat (Rubenthaler et al., 1990; Addo et al., 1991; Slade and Levine, 1994; Kweon et al., 2011; Liu et al., 2017b), which can be measured by various tests such as sodium dodecyl sulfate (SDS)-sedimentation volume (SSV), extensograph, farinograph, alveograph, and gluten index (Huang et al., 2006; Elangovan et al., 2008; Li et al., 2009; Kerfal et al., 2010; Tsilo et al., 2011). The SSV test is well correlated with gluten strength and bread-making quality of wheat (Axford et al., 1979; He et al., 2004; Ozturk et al., 2008), and exhibits advantages such as simplicity, low cost, small sample size requirement, and high efficiency. Therefore, it has been widely used for evaluating the content and quality of gluten protein and for fast screening desired cultivars in wheat breeding programs (Clarke et al., 2000).

Some recent studies have found that the quantitative nature of SSV is closely correlated to multiple genes encoding glutenins and gliadins, such as *Glu-1*, *Glu-A3*, *Glu-B3*, and *Gli-B1* (Payne and Lawrence, 1983; Payne et al., 1984; Shewry et al., 2003; Maucher et al., 2009; Reif et al., 2011; Deng et al., 2015; Guo et al., 2020). Glutenins and gliadins are not only the most important storage proteins of wheat but also the main components of gluten protein (Gianibelli et al., 2001; Kerfal et al., 2010). Glutenins are related to the extensibility of gluten, while gliadins are associated with the elasticity of gluten (MacRitchie, 1995; Veraverbeke and Delcour, 2002; Van Der Borght et al., 2005; Rasheed et al., 2014). The content and ratio of glutenins and gliadins are the main factors that determine the wheat processing quality (Yang et al., 2014). In addition, puroindolines are a component of wheat grain protein and are closely related to grain hardness. *Puroindoline b (Pinb-D1) gene* was found to be related to the variation of SSV in recent studies (Park et al., 2010, 2012; Ahn et al., 2014; Würschum et al., 2016).

Sodium dodecyl sulfate (SDS)-sedimentation volume (SSV) is a complex quantitative trait affected by both environmental and genetic factors. Quantitative trait locus (QTL) analysis is an effective approach for examining the genetic basis of quantitative traits (Doerge, 2002). Many studies have analyzed QTLs for SSV. To date, QTLs for SSV have been detected on almost all chromosomes, explained 2.2–41.4% of the phenotypic variation (Li et al., 2009; Kerfal et al., 2010; Reif et al., 2011; Deng et al., 2015; Würschum et al., 2016; Liu et al., 2017a; Mir Drikvand et al., 2018; Goel et al., 2019; Guo et al., 2020; Yang et al., 2020). However, most of the previously identified QTLs were detected only in one or two environments and could not be detected in multiple genetic backgrounds, which were not ideal targets for fine mapping and map-based cloning.

Here, to understand the genetic basis underlying SSV and provide molecular markers linked to QTL for wheat quality breeding, a doubled haploid (DH) population derived from a cross between Non-gda3753 (ND3753) and Liangxing99 (LX99) was employed to detect the QTLs associated with SSV variation. The genetic effect of two major SSV QTLs was validated.

## Materials and Methods

### Plant Materials

The DH population consisting of 123 individuals was developed through *in vitro* anther culture (De Buyser and Henry, 1980) of the F_1_ hybrids from a cross between ND3753 and LX99. The DH population and two parents were used for genome-wide identification of QTLs related to SSV.

For mapping of the possible QTL, ND3753 that carried the positive allele of the QTL in the confidence interval was crossed with LX99. F_1_ plants were backcrossed with LX99 for three generations with insertion-deletion (InDel) marker-assisted selection to generate a BC_3_F_1_ population containing 418 plants. Subsequently, 126 heterozygotes lines at the QTL-anchored region were self-pollinated to BC_3_F_2_ containing 1,081 plants. This population with LX99 background is presented as BC_3_F_2_-L in the present paper.

In addition, in order to evaluate the effects of two QTL, LX99 was crossed with recurrent parent ND3753 and 64 BC_3_F_1_ heterozygotes lines at the QTL-anchored region were self-pollinated to construct another BC_3_F_2_ population containing 387 plants with marker-assisted selection. This population with the background of ND3753 is presented as BC_3_F_2_-N.

### Field Trials

The DH population and two parents were planted in seven environments during the wheat-growing seasons of 2016, 2017, and 2018 in Beijing (BJ) (40°08′N, 116°10′E), Linfen (LF) (36°04′N, 111°31′ E), Xi’an (XA) (34°16′N, 108°55′E), and Cangzhou (CZ) (38°18′N, 116°49′E), China. The seven environments, namely 2016BJ, 2017BJ, 2017LF, 2017XA, 2018BJ, 2018LF, and 2018CZ were presented in this study as E1, E2, E3, E4, E5, E6, and E7, respectively. The field trials were conducted following a complete random block design with three biological replicates. However, only one biological duplication was harvested in E7 due to an accident of field management. Then 60 seeds for each of the lines and two parents were planted in two rows of 1.5 long and the row space was 20 cm.

The BC_3_F_1_, BC_3_F_2_-L, and BC_3_F_2_-N populations were all planted in Beijing. The BC_3_F_1_ population was planted in the wheat-growing seasons of 2018, while the BC_3_F_2_-L and BC_3_F_2_-N populations were planted in 2019. All of these backcross populations were sown in rows of 1.5 m long and 30 cm row space with a sowing density of 20 seeds per row. The BC populations were all planted in one trial and designed as a single replicate. During the whole growing season, the local standard field management methodologies were adopted for plant cultivation.

### Evaluation of Traits

For DHs, 80 plants were harvested in each line of the seven environments and wheat flour of each line planted in E1 was obtained with a CD1 Quadrumat Junior laboratory mill (Chopin Technology, Paris, France), while the whole wheat flour of each line in the other six environments was produced by an XF-98B experimental mill (Zhenxing Electromechanical Instrument Factory, Cangzhou, China). SSV was determined according to a modified protocol of Axford et al. (1979) and Preston et al. (1982) using 2 g of samples. The specific procedure of the SSV test was similar to that described by Li et al. (2009). In particular, SSVs of each DH line with only one biological duplication harvesting from E7 were measured. SSV of the BC_3_F_1_, BC_3_F_2_-L, and BC_3_F_2_-N populations was measured with whole wheat flour from a single plant.

In addition, 300 g of grains of 30 randomly selected DH lines based on the minimum sample required for the Pearson’s correlation coefficient calculated according to the formula provided by Mangard et al. (2007) and Chen et al. (2011) in E5 were ground into flour with a flour yield of approximately 60% in all samples. Their farinograph parameters (GB/ICC) were recorded by a Farinograph (DongFu JiuHeng, Beijing, China) to evaluate the correlation with SSV (Chicago, IL, United States) (ICC, 1996; Luo et al., 2018).

### Genetic Map Construction

Deoxyribonucleic acid (DNA) was extracted from fresh leaves of individual DH lines and two parents using the hexadecyltrimethy ammonium bromide (CTAB) method (Allen et al., 2006). The 15 K Axiom^®^ Wheat Breeder single-nucleotide polymorphism (SNP) Genotyping Array (China Golden Marker Co., Beijing, China) containing 13,947 SNP markers was used to genotype the DH population and parents. SNP markers with a missing data rate > 20% were removed, and the remaining polymorphic markers were used to construct a wheat genetic map based on the inclusive composite interval mapping (ICIM) method using IciMapping v4.1 (Chinese Academy of Agricultural Sciences, China) and MapChart v2.32 (Plant Research International, P.O. Box 16, 6700 AA Wageningen) (Voorrips, 2002). The physical locations of unique SNP markers were obtained from the International Wheat Genome Sequencing Consortium (Appels et al., 2018).

### Quantitative Trait Locus Mapping

The average value of SSV in each environment and the BLUP were employed for QTL analysis using inclusive composite interval mapping (ICIM^1^) method in software IciMapping v4.1 (Meng et al., 2015). A QTL with LOD ≥ 2.5 was defined as a significant QTL. The confidence intervals (±2 LOD away from the peaks of likelihood ratios) of several QTLs were coincident, which were preliminarily considered as the same QTL. In this study, the QTL that can be detected in three or more environments is defined as an environmentally stable QTL.

### Re-sequencing and InDel Markers Development

High-quality genomic DNA of ND3753 and LX99 was extracted to construct paired-end-sequencing libraries. According to the procedures described by Li et al. (2020), the parents were re-sequenced with an average sequencing depth of 6 × and paired-end reads of length 150 bp for two parents using the Illumina HiSeq X Ten platform (Illumina, California, United States), and the re-sequencing data were processed. The InDels were identified using the HaplotypeCaller module of the Genome Analysis Toolkit (GATK). The InDel markers were developed based on the sequence difference between the parents around the target region. Primer3 version 0.4.0^2^ was used to design the sequences of InDel primers.

Deoxyribonucleic acid (DNA) amplification was programmed for an initial 5 min at 94°C, then followed by 35 cycles of 30 s at 94°C, 30 s at 56°C, and 30 s at 72°C, and finally 5 min at 72°C. A 10 μL PCR reaction system was used, containing 5 μL of 2 × Taq PCR StarMix (GenStar, Beijing, China) (for PAGE), 1.5 μL of DNA template (about 50–100 ng), 1.5 μL of each InDel primer, and double-distilled H_2_O. The PCR products were analyzed on 8% non-denaturing polyacrylamide gels with silver staining.

### Validation and Mapping and Annotation of Putative Genes

Insertion-deletion (InDel) markers tightly linked to *QSsv.cau-1A.1.1* and *QSsv.cau-1A.1.2* were used to genotype DH, BC_3_F_1_, BC_3_F_2_-L, and BC_3_F_2_-N populations. The putatively annotated high confidence (HC) genes located between the flanking markers of *QSsv.cau-1A.1.1* were acquired based on the reference genome of *T. aestivum* cv. Chinese Spring^3^.

### Statistical Analysis

To conduct phenotypic statistical analysis and calculate correlation coefficients between SSV and farinograph parameters, IBM SPSS Statistics 21.0 (SPSS, Chicago, United States) was used. R software v3.6.2^4^ was used to perform the Shapiro-Wilk test across seven environments and the best linear unbiased prediction (BLUP), as well as to estimate the broad-sense heritability (*h2B*) following the formula: $h_{B}^{2}=\sigma_{g}^{2}/\left(\sigma_{g}^{2}+\sigma_{g\,e}^{2}/n+\sigma^{2}/n\,r\right)$, as described by Liu et al. (2014).

## Results

### Phenotypic Analysis

**FIGURE 1:**
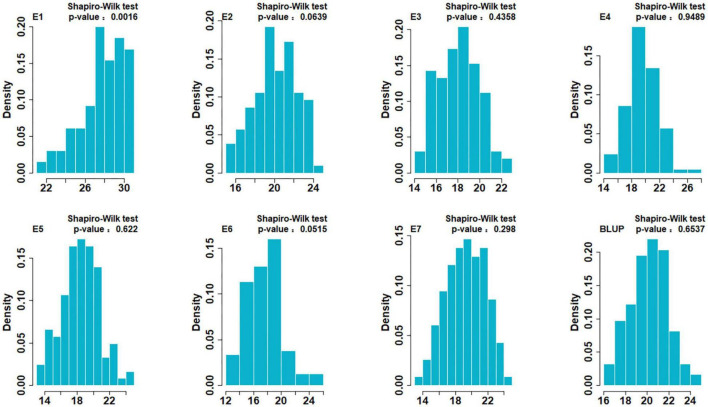
Histograms of the ND3753/LX99 DH population for SDS-sedimentation volume (SSV) under BLUP data. The Y-axis represents the density (the ratio of frequency to group distance) of each trait and the X-axis represents the phenotypic data.

Descriptive statistics for SSV of two parents and the DH population in the seven environments are shown in Table 1. SSV of ND3753 was significantly higher than that of LX99 in all environments. SSV showed bi-directional transgressive segregation, suggesting that both parents have increasing alleles for SSV. The broad-sense heritability values in all environments were greater than 0.8, indicating that SSV was mainly controlled by genetic factors. The result of the Shapiro-Wilk test displayed that SSV exhibited normal distribution under six environments and BLUP value, indicating SSV was determined by many genes (Figure 1). Pearson’s correlation coefficient analysis was carried out between farinograph parameters and SSVs of 30 DH lines planted in E5 which indicated a significant positive correlation between the two (Table 2).

**TABLE 1:** Descriptive statistics of two parents and DH population for SDS-sedimentation volume (SSV) under seven environments.

Trait	Environment^a^	Parents		DH population			
		ND3753	LX99	Range	Mean	SD^b^	*h2B* ^c^
SSV (mL)	E1	23.8 ± 0.9	20.0 ± 1.6	15.6–26.3	21.6	2.5	0.86
	E2	23.4 ± 2.6	19.4 ± 0.5	15.3–25.8	21.2	2.3	0.91
	E3	20.6 ± 0.8	15.8 ± 0.4	13.2–24.7	18.2	2.1	0.80
	E4	23.1 ± 1.4	16.4 ± 1.9	15.0–26.1	19.8	2.2	0.88
	E5	20.9 ± 3.3	17.2 ± 1.1	13.6–24.5	18.5	2.3	0.86
	E6	21.0 ± 0.4	14.2 ± 0.8	12.0–25.3	17.5	2.5	0.86
	E7	22.4	17.0	13.7–24.4	19.3	2.4	–

*^a^E1, 2016–2017 (Beijing); E2, 2017–2018 (Beijing); E3, 2017–2018 (Linfen); E4, 2017–2018 (Xi’an); E5, 2018–2019 (Beijing); E6, 2018–2019 (Linfen); E7, 2018–2019 (Cangzhou).*

*^b^SD, standard deviation.*

*^c^h2B, Broad sense heritability based on a family mean basis was estimated under individual environments.*

**TABLE 2:** Pearson’s correlation analysis among SDS-sedimentation volume (SSV), dough stability time (DST), dough developing time (DDT), and water absorption (WA) in E5/2018–2019 (Beijing).

Trait	SSV	DST	DDT	WA
SSV	1			
DST	0.614**	1		
DDT	0.595**	0.918**	1	
WA	0.536**	0.103	0.234	1

**Correlation is significant at the.01 level (2-tailed).

### Linkage Map Construction

A total of 2,523 SNP markers showed polymorphisms between the two parents. Finally, 2,474 SNP markers participated in the map construction and were mapped to 21 linkage groups, covering the 21 chromosomes of common wheat (Supplementary Table 1). The total length of the map was 7,349.01 cm, and the average interval distance between two adjacent markers was 7.24 cm (Supplementary Table 1). The A genome contained the most SNP markers (963), followed by the B genome (902), while the D genome had the least (609) (Supplementary Table 1). The total length of chromosome 7D was the largest (619.02 cm), while that of chromosome 4B was the smallest (180.86 cm) (Supplementary Table 1). Chromosome 5A harbored the most SNP markers (236), while chromosome 6D contained the least (42) (Supplementary Table 1).

### Quantitative Trait Locus Analysis

A total of 15 QTLs were detected on 11 chromosomes (1A, 1B, 1D, 2A, 2D, 4B, 4D, 5A, 5D, 6B, and 6D) in the seven environments (Table 3 and Supplementary Table 2). Four environmentally stable QTLs (*QSsv.cau-1A.1*, *QSsv.cau-1A.2*, *QSsv.cau-2D* and *QSsv.cau-5D.1*) were identified on chromosomes 1A, 1A, 2D, and 5D, respectively (Table 3). The favorable allele of *QSsv.cau-2D* came from LX99, while the superior alleles of the other three QTLs were contributed by ND3753. The major QTL *QSsv.cau-1A.1* was repeatedly detected in five environments and the BLUP data, explaining 39.67% of the phenotypic variation in the BLUP analysis (Table 3). *QSsv.cau-2D* and *QSsv.cau-5D.1* contributed 3.17 and 4.82% of the phenotypic variation in the BLUP analysis, respectively. *QSsv.cau-1A.2* explained 8.17–18.62% of the phenotypic variation. The remaining 11 were putative QTLs (Supplementary Table 2).

**TABLE 3:** The QTL regions harboring environmentally stable QTLs for SSV in the ND3753/LX99 DH population.

QTL	Environment	Flanking marker	Position (cM)	Interval (cM)	LOD	PVE^a^ (%)	Additive^b^
*QSsv.cau-1A.1*	E3	AX-109863151 and AX-110089093	164.0	163.1–166.1	6.15	13.69	0.83
	E4	AX-109863151 and AX-110089093	164.0	163.1–166.1	4.15	15.94	0.81
	E5	AX-109863151 and AX-110089093	164.0	163.1–166.1	7.40	19.70	0.88
	E6	AX-109863151 and AX-110089093	164.0	163.1–166.1	3.37	9.78	0.74
	E7	AX-109863151 and AX-110089093	164.0	163.1–166.1	4.99	12.14	0.77
	BLUP^c^	AX-109863151 and AX-110089093	164.0	163.1–165.1	23.65	39.67	1.32
*QSsv.cau-1A.2*	E1	AX-109863129 and AX-111450961	220.0	211.1–229.1	3.94	18.62	0.92
	E2	AX-109863129 and AX-111450961	218.0	213.1–224.1	8.42	21.03	1.01
	E5	AX-110673287 and AX-111688135	231.0	223.1–232.1	3.43	8.17	0.56
*QSsv.cau-2D*	E2	AX-110872666 and AX-110773527	143.0	142.9–143.9	3.19	7.15	–0.57
	E5	AX-111430851 and AX-110773527	144.0	143.9–148.9	4.87	12.20	–0.68
	E7	AX-110773527 and AX-109246010	153.0	151.9–155.9	5.79	13.75	–0.81
	E6	AX-110773527 and AX-109246010	155.0	151.9–155.9	4.74	13.56	–0.86
	BLUP	AX-110872666 and AX-110773527	143.0	142.9–143.9	2.91	3.17	–0.37
*QSsv.cau-5D.1*	E2	AX-89753391 and AX-109174882	501.0	495.9–506.9	7.30	18.31	0.92
	E4	AX-89753391 and AX-109174882	502.0	494.9–505.9	3.48	12.91	0.74
	E7	AX-89753391 and AX-109174882	501.0	490.9–509.9	3.18	7.40	0.60
	BLUP	AX-89753391 and AX-109174882	502.0	493.9–505.9	4.32	4.82	0.46

*^a^PVE, Phenotypic variation explained by the stable QTLs.*

*^b^Additive, the additive effect of a QTL, positive values: a favorable allele from ND3753; negative values: a favorable allele from LX99.*

*^c^BLUP, phenotype values based on best linear unbiased prediction.*

### Verification of *QSsv.cau-1A.1*

**FIGURE 2:**
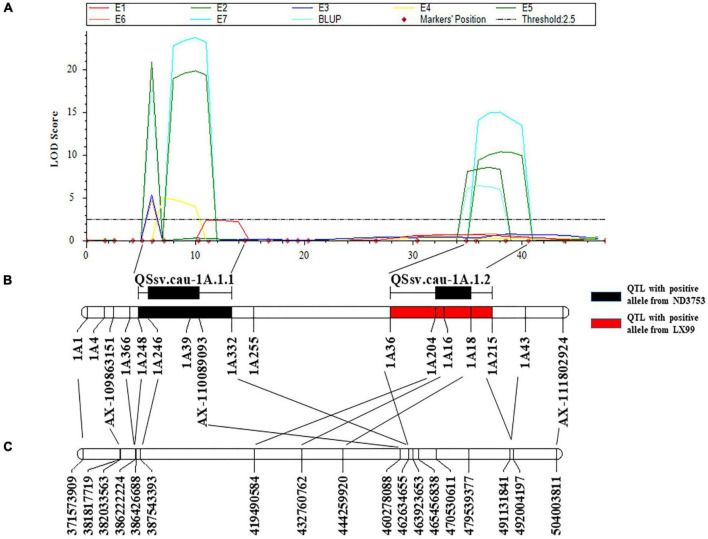
Genetic and physical locations of *QSsv.cau-1A.1.1* and *QSsv.cau-1A.1.2*. **(A)** QTL mapping for SSV in seven individual environments (E1–E7) and BLUP using a saturated genetic map of chromosome arm 1AL. **(B)** Saturated genetic map of chromosome arm 1AL with newly integrated InDel markers in the DH population. The black and red rectangles indicate QTLs with positive alleles from the parent ND3753 and the parent LX99, respectively. The black horizontal lines and bars above the genetic map represent the confidence interval of the two QTLs. **(C)** Corresponding physical positions according to the Chinese Spring IWGSC RefSeq v1.0 sequence.

Considering its stability and the genetic effect, *QSsv.cau-1A.1* was chosen to saturate the positioning interval. Then 15 InDel markers near this region were developed according to the re-sequencing results of two parents (Table 3 and Supplementary Table 3) and a new genetic linkage map of 1A long arm was constructed. QTL remapping detected an additional QTL located next to the original interval. These two QTLs were named *QSsv.cau-1A.1.1* and *QSsv.cau-1A.1.2* (Figure 2).

*QSsv.cau-1A.1.1* was detected in six environments and BLUP, explaining 17.21–26.47% of the phenotypic variation, and the favorable allele was from ND3753. The confidence interval was between the markers *1A248* and *1A332* corresponding CS physical position of 386,222,224–463,923,653 bp (Appels et al., 2018; Figure 2 and Supplementary Table 5). *QSsv.cau-1A.1.2* was repeatedly detected in three environments as well as BLUP data, contributing 7.02–12.13% of the phenotypic variation and LX99 contributed the favorable allele. The physical position of *QSsv.cau-1A.1.2* located on 462,634,655–492,004,197 bp by comparing flanking markers *1A36 and 1A215* to CS RefSeqv1.0 (Appels et al., 2018; Figure 2 and Supplementary Table 5).

### Effects of *QSsv-cau-1A.1.1* and *QSsv-cau-1A.1.2* in Different Genetic Backgrounds

The flanking markers *1A248*, *1A332* and *1A36*, *1A215* delimiting confidence intervals of *QSsv.cau-1A.1.1* and *QSsv.cau-1A.1.2* (Figure 2) separately were used to detect genotypes in the DH and BC_3_F_2_-N populations. AA and aa represented genotypes with homozygous favorable and unfavorable alleles of *QSsv.cau-1A.1.1* from ND3753, respectively, whereas BB and bb were symbols of that of *QSsv.cau-1A.1.2* from LX99.

**FIGURE 3:**
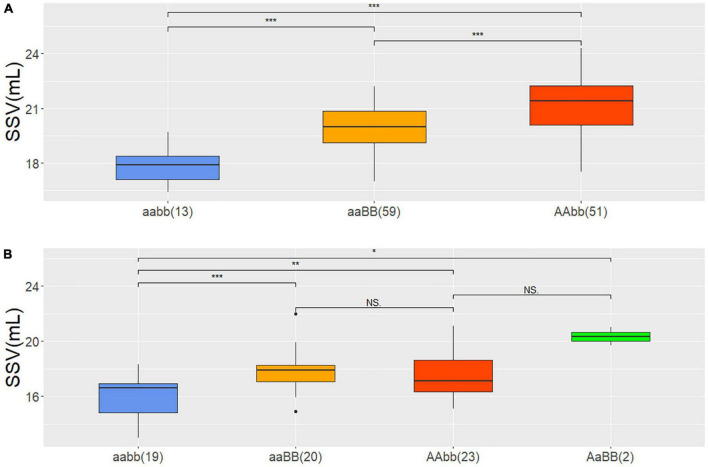
The phenotypic effect of *QSsv.cau-1A.1.1* and *QSsv.cau-1A.1.2* in the DH and BC_3_F_2_-N populations according to the BLUP value for SSV and the means of SSVs of different types, respectively. **(A)** DH population; **(B)** BC_3_F_2_-N population; *, **, and *** indicate significant differences at the.05, 0.01, and.001 levels, respectively (Student’s *t*-test). aabb: *QSsv.cau-1A.1.1* carrying homozygous alleles from LX99 and *QSsv.cau-1A.1.2* carrying homozygous alleles from ND3753; aaBB: *QSsv.cau-1A.1.1* carrying homozygous alleles from LX99 and *QSsv.cau-1A.1.2* carrying homozygous alleles from LX99; AAbb: *QSsv.cau-1A.1.1* carrying homozygous alleles from ND3753 and *QSsv.cau-1A.1.2* carrying homozygous alleles from ND3753; AaBB: *QSsv.cau-1A.1.1* carrying heterozygous alleles and *QSsv.cau-1A.1.2* carrying homozygous alleles from LX99. The numbers in parentheses indicate sample size.

DH lines could be grouped into three genotypes which included two parental genotypes AAbb, aaBB, and one recombined genotype aabb, and each contained 51, 59, and 13 lines. The reason for the absence of genotype AABB remained unknown. The average SSV values in BLUP of genotype AAbb, aaBB, and aabb were 21.2, 19.9, and 17.8 mL, respectively, of which AAbb was significantly higher than aaBB and both were significantly higher than that of aabb (Figure 3A). This suggested that *QSsv.cau-1A.1.1* had a stronger effect on SSV than *QSsv.cau-1A.1.2*, which was consistent with their contribution rates of phenotypic variation and additive effects in QTL analysis.

In BC_3_F_2_-N population four allele combinations AaBB, AAbb, aaBB, and aabb were identified, which had SSV average values of 20.4, 17.5, 17.9, and 15.8 mL from 2, 23, 20, and 19 plants, respectively. Similarly, the SSV values of genotypes AaBB, AAbb, and aaBB were significantly higher than that of aabb (Figure 3B). However, although genotype AaBB had the distinct highest value, three genotypes with favorable alleles had no significant difference. This was possibly due to the too-small sample number of genotypes AaBB. Nevertheless, all results above could still prove that the favorable allele has positive effects.

This study did not find the combination type of AABB but found the type AaBB in the BC_3_F_2_-N population. In summary, the combination of two favorable loci had the high SSV, whereas recombinants with unfavorable loci had the lowest. In future research, the homozygous lines of the BC_3_F_2_-N population will be extracted to develop the corresponding NIL pairs to further verify their effects and examine their genetic effect on some wheat qualities, such as gluten content, extensograph, farinograph, alveograph, and gluten index.

### Mapping of *QSsv-cau-1A.1.1*

**FIGURE 4:**
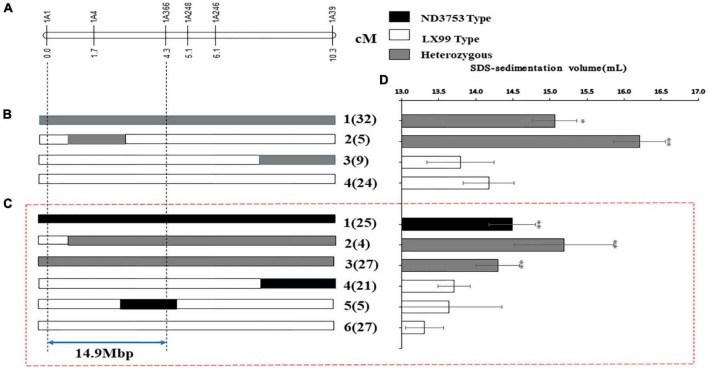
Mapping of *QSsv-cau-1A.1.1.* **(A)** Genetic location of the region of interest on chromosome arm 1AL. Graphical illustration of recombinant genotypes from **(B)** the BC_3_F_1_ population and **(C)** the BC_3_F_2_ population in the LX99 background. **(D)** SSV values (mean ± SD). Black, gray, and white bars represent the ND3753 genotype, heterozygous genotype, and the LX99 genotype, respectively. The arrow indicates the 14.9-Mb mapping interval. Significant differences by comparing with the ND3753 genotype are indicated by * (*p* < 0.05), and ** (*p* < 0.05) (Student’s *t*-test). The numbers in and outside the parentheses indicate sample size and recombinant types, respectively.

In order to further verify and narrow down the confidence interval of *QSsv.cau-1A.1.1*, the BC_3_F_1_ population in the background of LX99 was genotyped using six InDel markers, and four recombinant types were obtained (Figure 4B). The SSV values of types 3 and 4 were similar and were significantly lower than that of types 1 and 2, indicating that *QSsv.cau-1A.1.1* was delimited to the interval between markers *1A1* and *1A366*.

The BC_3_F_1_ individuals with heterozygous genotypes between markers *1A1* and *1A39* were selected to generate the BC_3_F_2_-L population. The six markers between *1A1* and *1A39* were used to genotype the BC_3_F_2_-L population, and six recombinant types were obtained (Figure 4C). The SSV value of type 6 was similar to that of types 4 and 5 but was significantly lower than that of the other types (Figure 4D). These results once again proved the effectiveness of *QSsv.cau-1A.1.1* and further confirmed that its location was between markers *1A1* and *1A366* (Figure 4C). There was no significant phenotypic difference between the heterozygous genotype and the ND3753 genotype, which implied that the ND3753 genotype was dominant. The corresponding physical interval of CS between these two markers was 14.9 Mb, with 89 putative high-confidence annotated genes (Supplementary Table 6). In addition, further fine mapping of *QSsv-cau-1A.1.1* is under research.

## Discussion

### Correlation of Sodium Dodecyl Sulfate-Sedimentation Volume and Farinograph Parameters

Sodium dodecyl sulfate (SDS)-sedimentation volume (SSV) is a comprehensive indicator for indirectly testing wheat quality and one of the important tests to evaluate the gluten strength of flour and is closely related to the processing and baking quality of flour (Axford et al., 1978; Peña-Bautista, 2002; He et al., 2004). SSV is well correlated with other quality traits, such as grain protein content, gluten index, wet gluten content, bread volume, and farinograph parameters (Cubadda et al., 1992).

Our results showed that SSV was significantly positively correlated with stability time, formation time, and water absorption of dough, which is consistent with previous studies (Table 2; Cubadda et al., 1992). This confirms that SSV can be used as a suitable substitute for farinograph indicators that require a great number of samples to evaluate the rheological properties of wheat dough, thereby indirectly measuring the baking and processing quality of wheat flour.

### Novel Quantitative Trait Locus for Sodium Dodecyl Sulfate-Sedimentation Volume

We compared the physical locations of QTLs for SSV reported in previous studies and those revealed in this study (Li et al., 2009; Reif et al., 2011; Deng et al., 2015; Würschum et al., 2016; Yang et al., 2020). The physical position of *QSsv.cau-1A.2* was agreed with that of a previously reported QTL controlling SSV (Yang et al., 2020). SSV was found to be affected by allelic variations at *Glu-A1* (508,726,618–508,725,448 bp, RefSeqv1.0) and *Glu-A3* (4,203,001–4,202,275 bp, RefSeqv1.0) loci in several previous studies (Li et al., 2009; Reif et al., 2011; Deng et al., 2015; Würschum et al., 2016). Some QTLs associated with SSV was reported on chromosome 1A. For instance, Yang et al. (2020) identified a QTL (540,660,000–544,610,000 bp, RefSeqv1.0) for SSV that is located on chromosome 1A by genome-wide association study (GWAS). However, the physical position of *QSsv.cau-1A.1.1* (371,573,909–386,426,688 bp, RefSeqv1.0) and *QSsv.cau-1A.1.2* (419,490,584–492,004,197 bp, RefSeqv1.0) did not overlap with those of the above-mentioned QTLs/genes, suggesting these two QTLs may be novel. Li et al. (2009) identified a QTL (470,230,000–570,420,000 bp, RefSeqv1.0) for SSV on chromosome 2D using a recombinant inbred line population. Four QTLs (16,340,000, 59,102,000, 615,470,000, 646,600,000 bp, RefSeqv1.0) controlling SSV were reported to be located on chromosome 2D by multi-locus GWAS (Yang et al., 2020). However, the physical position of these QTLs and that of *QSsv.cau-2D* (140,759,212–467,689,413 bp, RefSeqv1.0) were not consistent, indicating that *QSsv.cau-2D* may also be a novel QTL. Li et al. (2009) identified a QTL for SSV on chromosome 5DS. SSV was found to be affected by the allelic variation at the *Pinb-D1* locus on chromosome 5DS in some previous studies (Li et al., 2009; Reif et al., 2011; Deng et al., 2015; Würschum et al., 2016). However, the physical locations of these QTLs/genes and *QSsv.cau-5D.1* does not match, implying that *QSsv.cau-5D.1* on chromosome 5DL may be a novel QTL.

Sodium dodecyl sulfate (SDS)-sedimentation volume (SSV) is a quantitative trait affected by both environmental and genetic factors; thus, some QTLs can only be detected in specific environments (Supplementary Table 2). We found 11 such QTLs located on chromosomes 1B, 1D, 2A, 4B, 4D, 5A, 5D, 6B, and 6D, which is consistent with previous results (Li et al., 2009; Kerfal et al., 2010; Reif et al., 2011; Deng et al., 2015; Würschum et al., 2016; Liu et al., 2017a; Mir Drikvand et al., 2018; Goel et al., 2019; Guo et al., 2020; Yang et al., 2020). The *Glu-D1* gene, which is located in the interval of *QSsv.cau-1D*, may be a candidate gene for *QSsv.cau-1D*. *QSsv.cau-4B.1* and *QSsv.cau-4D* was located next to the dwarf genes *Rht-B1* and *Rht-D1*, respectively. Previous studies have also revealed QTL-enrichment areas near *Rht-B1* and *Rht-D1*, which are associated with kernel size, kernel hardness, kernel protein, pasting properties, and mixing properties (Shanhong et al., 2001; Li et al., 2006; Wang et al., 2012, 2017; Patil et al., 2013; Zhang et al., 2013; Jin et al., 2016; Liu et al., 2017b).

However, there is not enough evidence to support the correlation between *Rht-B1/D1* and SSV, and the gene that controls SSV near *Rht-B1* and *Rht-D1* has not been cloned. We hypothesized: (1) there may be other genes affecting quality traits near *Rht-B1* and *Rht-D1*; (2) allelic variations between *Rht-B1* and *Rht-D1* may also regulate certain quality traits, such as SSV. However, these hypotheses need to be further tested. The co-localization of dwarf genes and QTLs related to quality traits may also remind breeders to consider the selection of plant height and grain quality in the wheat breeding program.

### Genetic Effects and Putative Annotated Genes of the Major Quantitative Trait Locus

In recent years, a large number of QTLs for SSV have been identified and characterized through GWAS and linkage analysis, and some of these QTLs are related to allelic variants of *Glu-1*, *Glu-A3*, *Glu-B3*, *Gli-B1*, and *Pina-D1* (Ahn et al., 2014; Deng et al., 2015; Guo et al., 2020). However, most of the other QTLs have not been further verified or fine mapped. SSV is a typical quantitative trait with a complex genetic mechanism. The lack of information on the authenticity and genetic effects of these QTLs for SSV not only hinders the exploration of their genetic and molecular mechanisms but also fails to provide breeders with sufficient new high-quality genetic resources for wheat quality improvement. In this study, we verified the effects of *QSsv.cau-1A.1.1* and *QSsv.cau-1A.1.2* on SSV and the interaction between the two QTLs in the DH and BC3F2-N populations. Therefore, the InDel markers are closely linked to *QSsv.cau-1A.1.1* and *QSsv.cau-1A.1.2* developed in this study can be used by breeders to aggregate high-quality genes for wheat quality improvement.

In particular, *QSsv.cau-1A.1.1* was delimited to an approximate 14.9 Mb between markers *1A1* and *1A366* (Figure 4). We conduct an orthologous analysis for the candidate region to predict HC genes in *QSsv.cau-1A.1.1*, but no ones are associated with SSV in Oryza sativa and Arabidopsis thaliana (Supplementary Table 6). This may be because Oryza sativa and Arabidopsis thaliana do not have gluten, and SSV is related to gluten strength in the common wheat.

## Data Availability Statement

The raw data of Nongda3753 and Liangxing99 presented in the study are deposited in the NCBI Sequence Read Archive repository, accession number PRJNA722149.

## Author Contributions

MY conceived the project. ST performed the research, constructed the linkage map, developed an InDel marker of the QTL region of interest, and developed the BC_3_F_1_ and BC_3_F_2_ populations. JL, MZ, and SW participated in the field trials. ST, CB, HZ, CW, ZC, JY, and XS performed the phenotypic analysis. YZ, CX, BL, RL, and QS assisted in revising the manuscript. ST and YZ analyzed the experimental results. ST and MY wrote the manuscript. All authors contributed to the article and approved the submitted version.

## Conflict of Interest

The authors declare that the research was conducted in the absence of any commercial or financial relationships that could be construed as a potential conflict of interest.

## Publisher’s Note

All claims expressed in this article are solely those of the authors and do not necessarily represent those of their affiliated organizations, or those of the publisher, the editors and the reviewers. Any product that may be evaluated in this article, or claim that may be made by its manufacturer, is not guaranteed or endorsed by the publisher.

## Footnotes

1
http://www.isbreeding.net/software/?type=detail&id%20=18

2
http://bioinfo.ut.ee/primer3-0.4.0/

3
https://urgi.versailles.inra.fr/download/iwgsc/IWGSC_RefSeq_Annotations/v1.0/

4
https://www.r-project.org/
